# Supplementary material for: Drivers of Dyadic Cofeeding Tolerance in Pan: A Composite Measure Approach
Source: Biology (Basel). 2022 May 6;11(5):713. doi: 10.3390/biology11050713 (PMC9138277; doi:10.3390/biology11050713)
Supplement: Supplementary file 1 [file biology-11-00713-s001.zip › biology-1674787-supplementary.pdf]

Drivers of Dyadic Cofeeding Tolerance in *Pan*: A composite measure approach

Nicky Staes[(1,2)]*, Kim Vermeulen[(1,2,3)]*, Edwin J.C. van Leeuwen[(1,2,3)], Jonas Verspeek[(1,2)], Jonas R.R. Torfs[(1,2)], Marcel Eens[(1)] & Jeroen M.G. Stevens[(1,4)],

* Shared first authorship

Corresponding author: nicky.staes@uantwerpen.be

[1] Behavioural Ecology and Physiology Group, Department of Biology, University of Antwerp, Belgium

[2] Centre for Research and Conservation, Royal Zoological Society of Antwerp, Belgium

[3] Animal Behaviour and Cognition, Department of Biology, Utrecht University, the Netherlands

[4] SALTO Agro- and Biotechnology, Odisee University College, Belgium

Relationship quality variables

Table S1. Behavioural variables, with corresponding definitions, scored during the naturalistic observations to determine measures of relationship quality (see Stevens et al., 2015).

Behavioural variable	Definition
Grooming frequency	Number of grooming bouts exchanged within a dyad, i.e. the sum of all bouts from A to B and from B to A.
Grooming symmetry	Symmetry of grooming within a dyad A and B, calculated using the following formula: A grooms B/(A grooms B + B grooms A). For each dyad, the lowest of the two values obtained reversing A's and B's roles was chosen to represent the degree of symmetry (ranging from 0 to 0.5).
Proximity	Proportion of scans spent within arm's reach.
Aggression frequency	Frequency of all aggressive interactions within a dyad.
Aggression symmetry	Symmetry of aggression within a dyad (calculated in the same way as grooming symmetry).
Support	Index of agonistic support (frequency of support/opportunity to support). Support was defined as all instances where an individual A intervenes with an aggression within 30 sec in an agonistic interaction between two other individuals B and C to aid in attack or defence.
Counter-intervention	Index of counter-intervention (frequency of counter-intervention/opportunity to intervene). Every support pro for an individual implied contra support against a victim. The target of a coalition is considered as the receiver of counter-intervention.
Peering	Frequency of *peering* (= the subject stares at the receiver's face from a very close distance, up to a few centimetres) within a dyad.

Table S2. Varimax rotated factor loadings for the factors of relationship quality (RQ) in chimpanzees only. Boldface indicates high loadings ≥ |0.5|.

Variable	Relationship Incompatibility	Relationship Value
Aggression frequency	**0.993**	0.051
Aggression symmetry	**0.716**	0.119
Counter-intervention	**0.512**	0.080
Grooming frequency	0.100	**0.979**
Grooming symmetry	0.100	**0.659**
Proximity	0.282	**0.632**
Peering	-0.062	0.152
Support	-0.027	-0.066
% of variation explained	33.68%	20.51%
Eigenvalue	2.69	1.64

Table S3. Raw data cofeeding tolerance experiment in bonobos and chimpanzee

Sp	Gr	Dyad	Mat kin	Age	Sex	conflict	Tol food	Neg food	Prox	Tog plot	RV	RI	TC	AC	inplot
1	5	BYBS	0	14	FF	0.00	0.00	0.00	0.06	0.00	0.14	-1.10	0.18	-0.08	Yes
1	5	BYDJ	0	5	FF	0.00	0.00	0.00	0.28	0.22	0.23	1.51	0.42	-0.22	Yes
1	5	BSDJ	0	9	FF	0.00	0.00	0.00	0.11	0.00	0.36	0.58	0.23	-0.09	Yes
1	5	BYHB	0	16	FM	4.10	0.00	0.00	0.22	0.21	-0.14	3.23	0.33	0.22	Yes
1	5	BSHB	0	2	FM	16.40	0.00	0.00	0.06	0.00	1.47	3.17	-0.29	0.69	Yes
1	5	DJHB	1	11	FM	0.00	4.10	0.00	0.47	0.23	0.23	-0.98	0.56	-0.27	Yes
1	4	HAHR	0	6	FM	0.00	0.00	0.00	0.14	0.67	0.00	-0.01	0.46	-0.32	Yes
1	5	BYHO	0	12	FF	0.00	0.00	0.00	0.30	0.21	-0.02	-1.15	0.43	-0.22	Yes
1	5	BSHO	0	26	FF	0.00	0.00	0.00	0.14	0.00	0.34	-0.88	0.25	-0.10	Yes
1	5	DJHO	0	17	FF	0.00	0.00	0.00	0.56	0.61	0.25	0.64	0.63	-0.59	Yes
1	5	HBHO	0	28	FM	4.10	0.00	0.00	0.41	0.35	-0.86	0.55	0.48	0.18	Yes
1	4	HAKM	0	20	FF	0.00	0.00	0.00	0.14	0.78	0.35	-0.89	0.49	-0.37	Yes
1	4	HRKM	0	14	FM	0.00	0.00	0.00	0.17	0.64	0.30	-0.37	0.47	-0.32	Yes
1	5	BYKG	0	15	FF	0.00	0.00	0.00	0.05	0.00	0.62	-0.45	0.17	-0.07	Yes
1	5	BSKG	0	1	FF	0.00	0.00	0.00	0.05	0.00	1.76	-0.92	0.17	-0.07	Yes
1	5	DJKG	0	10	FF	0.00	0.00	0.00	0.09	0.33	-0.33	0.67	0.34	-0.18	Yes
1	5	HBKG	0	1	FM	0.00	0.00	0.00	0.09	0.33	1.02	-0.54	0.34	-0.18	Yes
1	5	HOKG	0	27	FF	0.00	0.00	0.00	0.11	0.14	0.24	-0.49	0.28	-0.13	Yes
1	4	HAMG	0	56	FF	0.00	0.00	0.00	0.09	0.00	-1.02	-0.84	0.21	-0.09	Yes
1	4	HRMG	0	50	FM	3.56	0.00	0.00	0.19	0.58	-0.57	-0.94	0.43	0.14	Yes
1	4	KMMG	0	36	FF	0.00	0.00	0.00	0.36	0.70	-0.06	-0.71	0.57	-0.47	Yes
1	3	BAMX	0	5	FF	0.00	0.00	0.00	0.10	1.00	1.58	-0.90	0.52	-0.46	Yes
1	4	HANT	0	43	FF	0.00	0.00	0.00	0.00	0.00	0.78	-0.92	0.12	-0.06	No
1	4	HRNT	1	37	FM	0.00	0.00	0.00	0.03	0.50	0.25	-0.66	0.35	-0.21	Yes
1	4	KMNT	0	23	FF	0.00	0.00	0.00	0.00	0.00	0.00	-0.73	0.12	-0.06	No
1	4	MGNT	0	13	FF	0.00	0.00	0.00	0.03	0.00	0.28	-0.67	0.15	-0.07	Yes
1	4	HANA	0	1	FF	0.00	0.00	0.00	0.00	0.00	0.99	-0.85	0.12	-0.06	No

1	4	HRNA	0	5	FM	0.00	0.00	0.00	0.03	0.50	-0.11	0.32	0.35	-0.21	Yes
1	4	KMNA	0	19	FF	0.00	0.00	0.00	0.44	0.46	-0.72	-0.93	0.55	-0.40	Yes
1	4	MGNA	0	55	FF	0.00	0.00	0.00	0.27	0.53	-0.26	-0.66	0.49	-0.33	Yes
1	4	NTNA	0	42	FF	0.00	0.00	0.00	0.02	1.00	-0.68	-0.90	0.48	-0.41	Yes
1	5	BYNY	0	22	FF	0.00	0.00	0.00	0.17	0.18	-1.04	-0.63	0.34	-0.16	Yes
1	5	BSNY	0	8	FF	0.00	0.00	0.00	0.09	0.17	0.04	-0.78	0.28	-0.13	Yes
1	5	DJNY	1	17	FF	0.00	0.00	0.00	0.28	0.50	2.07	-0.89	0.49	-0.32	Yes
1	5	HBNY	1	6	FM	20.50	0.00	12.30	0.20	0.31	0.03	0.54	0.10	1.05	Yes
1	5	HONY	0	34	FF	0.00	0.00	0.00	0.33	0.48	0.77	-1.08	0.51	-0.34	Yes
1	5	KGNY	0	7	FF	0.00	0.00	0.00	0.06	0.00	0.63	0.04	0.18	-0.08	Yes
1	3	BANY	0	1	FM	0.00	0.00	0.00	0.03	1.00	1.07	0.79	0.49	-0.41	Yes
1	3	MXNY	0	6	FM	0.00	0.00	0.00	0.28	0.45	1.01	-0.01	0.48	-0.30	Yes
1	3	BAPN	0	3	FM	5.46	0.00	0.00	0.00	0.00	-0.63	2.09	-0.01	0.35	Yes
1	3	MXPN	0	8	FM	0.00	0.00	0.00	0.10	0.00	-0.93	3.69	0.22	-0.09	Yes
1	3	NYPN	0	2	MM	0.00	0.00	0.00	0.08	0.00	-1.46	1.30	0.20	-0.08	Yes
1	3	BASA	0	6	FM	0.00	0.00	0.00	0.00	0.00	-1.12	1.54	0.12	-0.06	No
1	3	MXSA	0	11	FM	0.00	0.00	0.00	0.00	0.00	-1.20	1.81	0.12	-0.06	No
1	3	NYSA	0	5	MM	0.00	0.00	0.00	0.00	0.00	-0.56	-0.22	0.12	-0.06	No
1	3	PNSA	0	3	MM	0.00	0.00	0.00	0.00	0.00	0.09	-0.30	0.12	-0.06	No
1	5	BYVI	0	4	FM	0.00	0.00	0.00	0.00	0.00	-0.72	-0.69	0.12	-0.06	No
1	5	BSVI	0	10	FM	0.00	0.00	0.00	0.00	0.00	0.24	-0.63	0.12	-0.06	No
1	5	DJVI	0	1	FM	0.00	0.00	0.00	0.00	0.00	0.89	-0.92	0.12	-0.06	No
1	5	HBVI	0	12	MM	0.00	0.00	0.00	0.00	0.00	1.04	0.74	0.12	-0.06	No
1	5	HOVI	1	16	FM	0.00	0.00	0.00	0.00	0.00	0.06	-0.70	0.12	-0.06	No
1	5	KGVI	0	11	FM	0.00	0.00	0.00	0.00	0.00	-0.36	0.30	0.12	-0.06	No
1	5	NYVI	0	18	FM	0.00	0.00	0.00	0.00	0.00	0.10	-0.53	0.12	-0.06	No
1	5	BYZA	0	8	FM	0.00	0.00	0.00	0.00	0.00	-0.91	0.24	0.12	-0.06	No
1	5	BSZA	0	6	FM	0.00	0.00	0.00	0.00	0.00	-0.12	-0.40	0.12	-0.06	No
1	5	DJZA	0	3	FM	0.00	0.00	0.00	0.00	0.00	-1.16	1.37	0.12	-0.06	No
1	5	HBZA	0	8	MM	0.00	0.00	0.00	0.00	0.00	0.24	0.58	0.12	-0.06	No

1	5	HOZA	1	20	FM	0.00	0.00	0.00	0.00	0.00	0.78	-1.02	0.12	-0.06	No
1	5	KGZA	0	7	FM	0.00	0.00	0.00	0.00	0.00	-0.42	-0.43	0.12	-0.06	No
1	5	NYZA	0	14	FM	0.00	0.00	0.00	0.00	0.00	-0.54	1.77	0.12	-0.06	No
1	5	VIZA	1	4	MM	0.00	0.00	0.00	0.00	0.00	0.27	-0.13	0.12	-0.06	No
1	3	BAZM	0	8	FF	0.00	0.00	0.00	0.00	0.00	-0.36	-1.07	0.12	-0.06	No
1	3	MXZM	0	3	FF	0.00	0.00	0.00	0.25	0.80	1.38	-0.98	0.54	-0.46	Yes
1	3	NYZM	0	9	FM	0.00	0.00	0.00	0.03	0.00	-0.42	2.16	0.15	-0.07	Yes
1	3	PNZM	1	11	FM	0.00	0.00	0.00	0.03	0.00	0.89	-0.58	0.15	-0.07	Yes
1	3	SAZM	1	14	FM	0.00	0.00	0.00	0.03	0.00	0.70	-0.66	0.15	-0.07	Yes
2	1	CEDA	0	14	FM	3.09	6.18	0.00	0.03	0.00	0.10	-0.05	0.23	0.23	Yes
2	2	ACDE	0	4	FM	0.00	4.53	0.00	0.11	0.29	-0.85	1.03	0.40	-0.14	Yes
2	2	ACJG	0	15	FM	4.53	4.53	0.00	0.11	0.14	0.22	1.97	0.30	0.28	Yes
2	2	DEJG	0	11	MM	0.00	13.58	4.53	0.16	0.70	-0.14	0.45	0.61	0.41	Yes
2	1	CEJO	0	13	FF	0.00	0.00	0.00	0.03	0.00	-0.61	-0.93	0.15	-0.07	Yes
2	1	DAJO	0	1	FM	0.00	0.00	0.00	0.57	0.11	-0.64	-0.69	0.54	-0.31	Yes
2	1	CEJZ	0	27	FF	0.00	0.00	0.00	0.00	0.00	-1.29	0.96	0.12	-0.06	No
2	1	DAJZ	0	13	FM	0.00	0.00	0.00	0.00	0.00	-1.39	0.72	0.12	-0.06	No
2	1	JOJZ	0	14	FF	0.00	0.00	0.00	0.00	0.00	0.68	-1.02	0.12	-0.06	No
2	1	CEJU	1	26	FM	0.00	0.00	0.00	0.00	0.00	-0.19	0.48	0.12	-0.06	No
2	1	DAJU	0	12	MM	3.09	0.00	0.00	0.37	0.00	0.67	0.63	0.37	0.16	Yes
2	1	JOJU	0	13	FM	0.00	0.00	0.00	0.19	0.58	0.94	0.64	0.47	-0.31	Yes
2	1	JZJU	0	1	FM	0.00	0.00	0.00	0.00	0.00	-1.18	0.32	0.12	-0.06	No
2	1	CELE	0	1	FF	0.00	0.00	0.00	0.00	0.00	-0.75	-0.74	0.12	-0.06	No
2	1	DALE	0	15	FM	0.00	3.09	0.00	0.08	0.20	-0.14	-0.54	0.33	-0.12	Yes
2	1	JOLE	0	14	FF	0.00	0.00	0.00	0.02	0.00	-1.02	-0.47	0.14	-0.06	Yes
2	1	JZLE	0	28	FF	0.00	0.00	0.00	0.00	0.00	-0.99	0.52	0.12	-0.06	No
2	1	JULE	0	27	FM	0.00	0.00	0.00	0.00	0.00	-1.03	-0.31	0.12	-0.06	No
2	2	ACLI	0	15	FF	0.00	0.00	0.00	0.00	0.00	-1.01	-1.05	0.12	-0.06	No
2	2	DELI	0	11	FM	0.00	0.00	0.00	0.00	0.00	0.30	-0.09	0.12	-0.06	No
2	2	JGLI	0	0	FM	0.00	0.00	0.00	0.00	0.00	1.84	-0.84	0.12	-0.06	No

2	1	CELU	0	5	FM	0.00	0.00	0.00	0.00	0.00	-1.20	-0.61	0.12	-0.06	No
2	1	DALU	0	9	MM	0.00	0.00	0.00	0.00	0.00	-1.18	-0.43	0.12	-0.06	No
2	1	JOLU	0	8	FM	0.00	0.00	0.00	0.00	0.00	-1.20	-0.61	0.12	-0.06	No
2	1	JZLU	0	22	FM	0.00	0.00	0.00	0.00	0.00	-1.25	-0.61	0.12	-0.06	No
2	1	JULU	0	21	MM	0.00	0.00	0.00	0.00	0.00	-0.01	-1.04	0.12	-0.06	No
2	1	LELU	0	6	FM	0.00	0.00	0.00	0.00	0.00	-0.87	-0.83	0.12	-0.06	No
2	2	ACMR	0	17	FF	0.00	4.53	0.00	0.08	0.40	-0.07	-0.46	0.42	-0.16	Yes
2	2	DEMR	0	13	FM	0.00	4.53	0.00	0.14	0.33	-0.17	0.33	0.43	-0.17	Yes
2	2	JGMR	0	2	FM	0.00	0.00	0.00	0.11	0.14	-0.52	1.62	0.28	-0.13	Yes
2	2	LIMR	0	2	FF	0.00	0.00	0.00	0.00	0.00	0.08	0.29	0.12	-0.06	No
2	1	CEML	0	2	FF	0.00	0.00	0.00	0.00	0.00	0.45	0.07	0.12	-0.06	No
2	1	DAML	0	16	FM	0.00	3.09	0.00	0.05	0.00	0.26	1.05	0.23	-0.06	Yes
2	1	JOML	0	15	FF	0.00	0.00	0.00	0.05	0.00	1.30	-0.94	0.17	-0.07	Yes
2	1	JZML	0	29	FF	3.09	3.09	0.00	0.00	0.00	-0.08	0.14	0.14	0.22	Yes
2	1	JUML	0	28	FM	0.00	0.00	3.09	0.05	0.67	-0.40	0.26	0.41	0.27	Yes
2	1	LEML	0	1	FF	0.00	0.00	0.00	0.00	0.00	-0.72	-0.83	0.12	-0.06	No
2	1	LUML	0	7	FM	0.00	0.00	0.00	0.00	0.00	-0.46	-0.52	0.12	-0.06	No
2	2	ACMA	1	20	FF	0.00	0.00	0.00	0.09	0.33	0.69	-0.64	0.34	-0.18	Yes
2	2	DEMA	0	16	FM	0.00	22.64	4.53	0.17	0.55	-0.07	0.86	0.66	0.43	Yes
2	2	JGMA	0	5	FM	4.53	27.17	0.00	0.20	0.15	0.38	2.49	0.61	0.32	Yes
2	2	LIMA	0	5	FF	0.00	0.00	0.00	0.00	0.00	-0.12	-0.69	0.12	-0.06	No
2	2	MRMA	0	3	FF	0.00	0.00	0.00	0.11	0.43	2.08	0.96	0.38	-0.22	Yes
2	2	ACMI	0	16	FF	0.00	0.00	0.00	0.08	0.40	0.92	-0.88	0.35	-0.19	Yes
2	2	DEMI	0	12	FM	0.00	9.06	0.00	0.09	0.00	0.28	2.70	0.37	-0.04	Yes
2	2	JGMI	0	1	FM	13.58	4.53	0.00	0.09	0.67	0.17	2.46	0.35	0.60	Yes
2	2	LIMI	0	1	FF	0.00	0.00	0.00	0.00	0.00	0.56	-0.86	0.12	-0.06	No
2	2	MRMI	0	1	FF	0.00	0.00	0.00	0.05	0.67	1.81	0.85	0.41	-0.27	Yes
2	2	MAMI	0	4	FF	0.00	9.06	0.00	0.08	0.40	1.78	0.60	0.47	-0.14	Yes
2	1	CENA	0	7	FF	0.00	0.00	0.00	0.02	1.00	0.29	-1.25	0.48	-0.41	Yes
2	1	DANA	0	7	FM	0.00	0.00	0.00	0.02	0.00	0.58	-0.67	0.14	-0.06	Yes

2	1	JONA	0	6	FF	0.00	0.00	0.00	0.02	0.00	-0.27	-0.92	0.14	-0.06	Yes
2	1	JZNA	0	20	FF	0.00	0.00	0.00	0.00	0.00	-0.54	2.15	0.12	-0.06	No
2	1	JUNA	0	19	FM	0.00	0.00	0.00	0.00	0.00	-0.08	0.02	0.12	-0.06	No
2	1	LENA	0	8	FF	0.00	0.00	0.00	0.00	0.00	-0.62	-0.93	0.12	-0.06	No
2	1	LUNA	0	2	FM	0.00	0.00	0.00	0.00	0.00	0.37	-0.76	0.12	-0.06	No
2	1	MLNA	0	9	FF	0.00	0.00	0.00	0.00	0.00	0.34	-1.09	0.12	-0.06	No
2	2	ACPA	0	19	FF	0.00	0.00	0.00	0.00	0.00	-0.65	-0.82	0.12	-0.06	No
2	2	DEPA	0	15	FM	0.00	0.00	0.00	0.00	0.00	0.00	0.38	0.12	-0.06	No
2	2	JGPA	0	4	FM	0.00	0.00	0.00	0.00	0.00	-0.74	1.99	0.12	-0.06	No
2	2	LIPA	0	4	FF	0.00	0.00	0.00	0.00	0.00	-0.30	-0.81	0.12	-0.06	No
2	2	MRPA	0	2	FF	0.00	0.00	0.00	0.00	0.00	-0.74	-0.82	0.12	-0.06	No
2	2	MAPA	0	1	FF	0.00	0.00	0.00	0.00	0.00	-0.21	-0.81	0.12	-0.06	No
2	2	MIPA	0	3	FF	0.00	0.00	0.00	0.00	0.00	1.07	-0.72	0.12	-0.06	No
2	2	ACSE	0	23	FM	0.00	0.00	0.00	0.11	0.29	-0.35	2.03	0.34	-0.17	Yes
2	2	DESE	0	19	MM	0.00	9.06	0.00	0.20	0.08	1.44	0.42	0.45	-0.09	Yes
2	2	JGSE	0	8	MM	0.00	0.00	0.00	0.19	0.08	-0.65	2.19	0.32	-0.14	Yes
2	2	LISE	0	8	FM	0.00	0.00	0.00	0.00	0.00	0.25	-0.77	0.12	-0.06	No
2	2	MRSE	0	6	FM	0.00	0.00	0.00	0.09	0.17	-0.36	0.61	0.28	-0.13	Yes
2	2	MASE	0	3	FM	0.00	4.53	0.00	0.22	0.29	-0.10	1.99	0.46	-0.18	Yes
2	2	MISE	0	7	FM	0.00	4.53	0.00	0.09	0.50	0.14	2.33	0.45	-0.20	Yes
2	2	PASE	0	4	FM	0.00	0.00	0.00	0.00	0.00	-0.99	1.64	0.12	-0.06	No
2	1	CESO	0	2	FM	0.00	0.00	0.00	0.03	0.00	-0.64	-0.64	0.15	-0.07	Yes
2	1	DASO	0	16	MM	0.00	37.08	6.18	0.75	0.81	0.97	1.03	0.91	0.49	Yes
2	1	JOSO	0	15	FM	3.09	30.90	0.00	0.57	0.56	1.11	-0.46	0.80	0.13	Yes
2	1	JZSO	0	29	FM	0.00	0.00	0.00	0.00	0.00	-0.27	-0.48	0.12	-0.06	No
2	1	JUSO	0	28	MM	0.00	0.00	0.00	0.32	0.40	0.34	0.22	0.49	-0.30	Yes
2	1	LESO	0	1	FM	0.00	0.00	0.00	0.05	0.00	-0.40	-0.92	0.17	-0.07	Yes
2	1	LUSO	0	7	MM	0.00	0.00	0.00	0.00	0.00	-0.69	-0.20	0.12	-0.06	No
2	1	MLSO	0	0	FM	0.00	0.00	0.00	0.05	0.00	-1.08	-0.50	0.17	-0.07	Yes
2	1	NASO	0	9	FM	0.00	0.00	0.00	0.02	0.00	0.90	-0.55	0.14	-0.06	Yes

2	2	ACST	0	28	FM	0.00	0.00	0.00	0.09	0.00	0.46	2.86	0.21	-0.09	Yes
2	2	DEST	1	24	MM	0.00	0.00	4.53	0.22	0.00	0.43	-0.28	0.31	0.43	Yes
2	2	JGST	0	13	MM	9.06	4.53	4.53	0.17	0.27	-0.94	1.41	0.33	0.69	Yes
2	2	LIST	0	13	FM	0.00	0.00	0.00	0.00	0.00	0.11	-0.61	0.12	-0.06	No
2	2	MRST	0	11	FM	0.00	0.00	0.00	0.09	0.00	-0.27	0.76	0.21	-0.09	Yes
2	2	MAST	0	8	FM	0.00	0.00	0.00	0.17	0.18	-0.91	1.96	0.34	-0.16	Yes
2	2	MIST	0	12	FM	0.00	0.00	0.00	0.08	0.60	-0.37	1.89	0.41	-0.26	Yes
2	2	PAST	0	9	FM	0.00	0.00	0.00	0.02	0.00	-0.53	1.59	0.14	-0.06	Yes
2	2	SEST	0	5	MM	0.00	9.06	0.00	0.23	0.73	0.15	0.17	0.61	-0.32	Yes
2	2	ACWA	0	19	FM	0.00	0.00	0.00	0.03	0.50	0.30	0.87	0.35	-0.21	Yes
2	2	DEWA	0	15	MM	0.00	0.00	0.00	0.05	0.00	0.69	0.84	0.17	-0.07	Yes
2	2	JGWA	0	4	MM	4.53	9.06	0.00	0.05	0.33	-0.77	-0.05	0.39	0.28	Yes
2	2	LIWA	0	4	FM	0.00	0.00	0.00	0.00	0.00	-0.37	0.37	0.12	-0.06	No
2	2	MRWA	0	2	FM	0.00	0.00	0.00	0.06	0.00	0.37	1.93	0.18	-0.08	Yes
2	2	MAWA	0	1	FM	0.00	0.00	0.00	0.06	0.75	0.47	-0.83	0.44	-0.32	Yes
2	2	MIWA	0	3	FM	0.00	0.00	0.00	0.02	0.00	0.22	2.36	0.14	-0.06	Yes
2	2	PAWA	0	0	FM	0.00	0.00	0.00	0.00	0.00	-0.33	-0.51	0.12	-0.06	No
2	2	SEWA	0	4	MM	0.00	0.00	0.00	0.05	0.33	1.93	1.80	0.31	-0.16	Yes
2	2	STWA	0	9	MM	0.00	0.00	0.00	0.05	0.33	-0.82	1.49	0.31	-0.16	Yes
2	1	CEWO	0	17	FM	0.00	0.00	0.00	0.03	0.50	-0.24	-1.00	0.35	-0.21	Yes
2	1	DAWO	0	3	MM	0.00	3.09	0.00	0.65	0.39	0.19	1.10	0.64	-0.47	Yes
2	1	JOWO	0	4	FM	0.00	9.27	0.00	0.41	0.04	-0.13	0.20	0.54	-0.15	Yes
2	1	JZWO	0	10	FM	0.00	0.00	0.00	0.00	0.00	-0.72	-0.83	0.12	-0.06	No
2	1	JUWO	0	9	MM	0.00	0.00	0.00	0.33	0.05	-0.01	-0.10	0.40	-0.18	Yes
2	1	LEWO	0	18	FM	0.00	0.00	0.00	0.06	0.00	-1.15	-0.60	0.19	-0.08	Yes
2	1	LUWO	0	12	MM	0.00	0.00	0.00	0.00	0.00	-1.05	-0.60	0.12	-0.06	No
2	1	MLWO	0	19	FM	0.00	0.00	0.00	0.05	0.33	-0.27	-1.26	0.31	-0.16	Yes
2	1	NAWO	0	10	FM	0.00	0.00	0.00	0.02	0.00	0.28	0.65	0.14	-0.06	Yes
2	1	SOWO	0	19	MM	3.09	9.27	0.00	0.63	0.23	1.31	-0.13	0.64	0.10	Yes

Sp = species (1=bonobo, 2=chimpanzee); Gr = Group; Mat kin = Maternal kinship; Tol food = tolerant food behaviors; Neg food = negative food behaviors; RV = Relationship Value; RI = Relationship Incompatibility; TC = Tolerant Cofeeding component; AC = Agonistic Cofeeding component; inplot refers to whether a dyad was seen to enter the plot during any of the experimental sessions

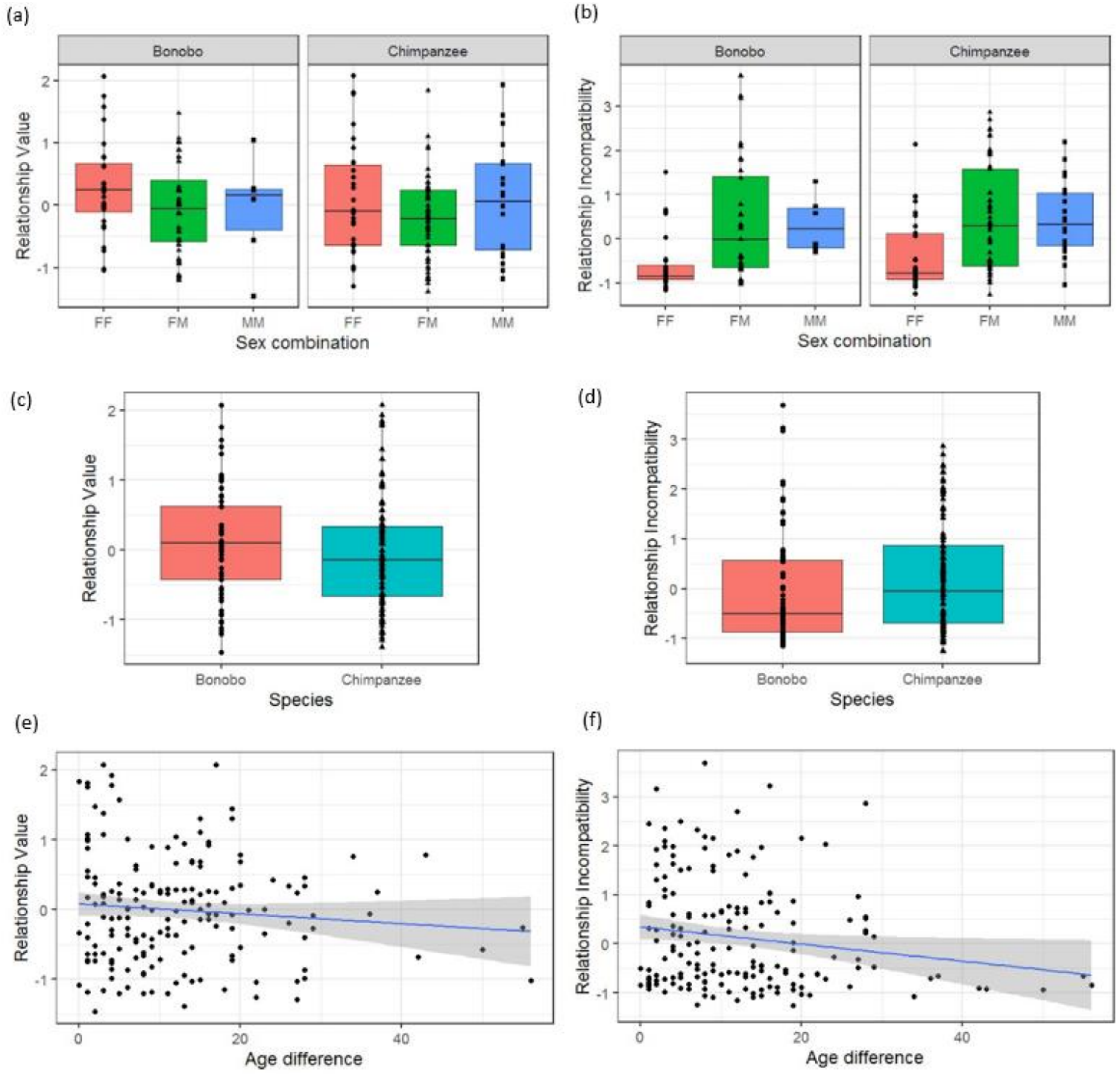

Figure S1. Relationship Quality components a) Relationship Value and b) Relationship Incompatibility by sex combination, where FF indicates female-female dyad, FM indicates female-male dyad and MM indicates male-male dyad, by species (c and d), and with age (e and f).
